# Predictors of Requirement of Inotrope Among Patients With Early Sepsis: Special Reference to Microcirculatory Parameters

**DOI:** 10.7759/cureus.24762

**Published:** 2022-05-05

**Authors:** Rishabh Bose, Gyanendra Singh, Prachi Singh, Ananyan Sampath, Ritik Singh, Bhupeshwari Patel, Abhijit P Pakhare, Rajnish Joshi, Sagar Khadanga

**Affiliations:** 1 Internal Medicine, All India Institute of Medical Sciences, Bhopal, IND; 2 Community and Family Medicine, All India Institute of Medical Sciences, Bhopal, IND; 3 Trauma (Paediatrics), All India Institute of Medical Sciences, Bhopal, IND

**Keywords:** handheld vital microscope, microcirculation, sublingual microscopy, predictors of sepsis, early sepsis

## Abstract

Introduction

The management of septic shock and refractory septic shock is essential in preventing sepsis-related death. The handheld vital microscope is a new modality of investigation for sepsis for microcirculatory assessment. This study aimed to identify predictors of inotrope requirements among patients with early sepsis and impending septic shock with particular reference to sublingual microcirculation assessment parameters.

Methodology

We conducted an observational cross-sectional hospital-based study in central India. The formal sample size was calculated to be 52 patients using a convenient sampling technique. The study was initiated with ethics approval (IHEC-LOP/2019/ MD0090) with consent from the patients. We used the MicroScan (MicroVision Medical, Netherlands) Video Microscope System (No.16A00102) to obtain sidestream dark-field imaging along with the AVA 4.3C software (MicroVision Medical).

Results

Of 51 cases, 60.8% were women, and 39.2% were men, and the study population had a mean age of 41.0 ± 14.9 years. Patients were recruited from medical wards (64.7%) and emergency departments (35.3%). The most common site of infection was gastrointestinal (33.3%), followed by respiratory infections (25.5%) and genitourinary infections (11.8%). The quick sequential organ failure assessment score was 2.0 ± 0.1. Eight patients required inotropes, and six patients died. High respiratory rates and lactate levels were important predictors of inotrope requirements in patients with early sepsis. Sublingual microcirculatory parameters at baseline did not significantly affect the requirement of inotropes consequently.

Conclusions

Sublingual microscopy is a suggested tool for the management of sepsis. However, without clearly defined cut-off values, handheld vital microscopy could not predict fluid responsiveness among patients with early sepsis. Also, it would be difficult to incorporate this technology into regular practice without equipment upgrades and image acquisition software.

## Introduction

Sepsis is a life-threatening organ dysfunction due to a dysregulated host response to infection [1,2]. A staggering 48.9 million cases of sepsis and 11 million sepsis-related-deaths were reported in 2017 [3]. Septic shock is the subset of sepsis in which cellular and metabolic abnormalities are profound enough to cause circulatory disharmony with low blood pressure [4-6]. Refractory shock combines shock and end-organ damage, requiring vasopressor support with a mortality of up to 60% [7].

Organ dysfunction is an important part of sepsis, and its quantification is essential for diagnosis, management, and prognostication. Globally, the sequential organ failure assessment (SOFA) score is one of the most used scores in sepsis to quantify organ dysfunction [8]. However, the SOFA score is challenging to calculate, needs specialized investigations, and takes time. The quick SOFA (qSOFA) is a simple bedside score to assess sepsis. It includes systolic blood pressure (SBP), respiratory rate, and altered sensorium as its three components. It is quick, easy to calculate, and helps in triaging patients with possible sepsis [8,9].

The 2021 Surviving Sepsis management guidelines advocate immediate resuscitation with intravenous crystalloids and the administration of broad-spectrum antibiotics, source control of the infection, and sending appropriate laboratory samples for diagnostics. The guidelines also emphasize the use of dynamic measures of fluid response (e.g., arterial pulse pressure variation) rather than static measures (e.g., pulse and blood pressure) while assessing response to fluid resuscitation [10]. Microvascular dysfunction and the accompanying tissue hypoxia result from the inflammatory sepsis cascade [2]. Conventionally macrocirculatory variables like pulse, systolic blood pressure (SBP) and diastolic blood pressure (DBP), mean arterial pressure (MAP), and central venous pressure are commonly used to assess the adequacy of the circulation and tissue hypoxia. However, these variables often fail to represent the microcirculatory status [11,12]. There is a lack of coherence between macro and microcirculatory variables, and the improvement of macrocirculatory variables does not go hand-in-hand with the microcirculatory status of the patient [13-15]. Even after an adequate amount of intravenous fluid has been transfused, some patients do not maintain optimum macrocirculatory parameters (e.g., SBP, DBP, MAP) and must ultimately be transfused with inotrope according to the guidelines [16]. However, critical time is lost to identifying whether the patient will be fluid responsive or need inotrope. There is an urgent need to monitor microcirculatory dynamic variables in the peripherally accessible blood vessels in this context.

Handheld vital microscopes (HVMs) can directly visualize the microcirculation at the nail bed and dermal capillaries, and they are used to assess various shock states and monitor patients after cardiovascular surgery [17-20]. HVMs seem to be a fascinating and promising new modality of investigation in the management of sepsis and septic shock [11,12,21]. In 2018, the European Society of Intensive Care Medicine (ESICM) task force advocated for sublingual microcirculation parameters like the proportion of perfused vessels (PPV) and the De Backer score to assess fluid administration in patients with sepsis and septic shock. The ESICM also emphasizes the need for identifying critical threshold values for various microcirculatory parameters for better classification of disease severity and identifying therapeutic endpoints [22]. HVMs are a potential option for the early identification of patients with early sepsis who will not improve with intravenous fluids alone and would ultimately require inotrope support. This will save precious time during patient management and potentially eliminate tissue hypoxia and the subsequent vicious sepsis-related inflammatory cascade. We conducted the present pilot study to assess the use of HVMs in early sepsis patients in a resource-limited busy setting-an Asian subcontinental public health care delivery center. 

## Materials and methods

We conducted this observational cross-sectional study on hospitalized patients at the All India Institute of Medical Sciences in Bhopal from November 2019 to April 2021. The study was approved by the Institutional Human Ethics Committee of All India Institute of Medical Science, Bhopal (Ref: IHEC-LOP/2019/ MD0090). All participants provided written informed consent to participate. The study was briefly interrupted due to the coronavirus disease 2019 pandemic during its first wave (April 2020 to October 2020) and second wave (March 2021 to April 2021). For sample size estimation, we assumed a population size of 1,00,000 and an anticipated frequency of fluid-unresponsive shock to be 5%, a confidence interval of 95%, and a design effect of 0.7. The sample size was calculated to be 52 using Open Source Epidemiologic Statistics for Public Health (OpenEpi) [23].

We included patients aged 18 or older with illness lasting <14 days with suspected infection and patients with SBP <100 mmHg but MAP >55mmHg and qSOFA score ≥ 2. Patients requiring intensive life support ventilated patients and patients who had received inotrope within 24 hours were excluded.

We administered a structured questionnaire and recorded the demographic, clinical, and laboratory parameters from the patients that determine the presence or severity of sepsis (i.e., body temperature, pulse rate, blood pressure, Glasgow Coma Scale (GCS) score, oxygen saturation, leukocyte count, serum lactate levels, platelet count, serum creatinine, total bilirubin, blood urea, aspartate aminotransferase, alanine transaminase, pH, partial pressure of carbon dioxide, and other arterial blood gasses parameters).

We used the MicroScan (MicroVision Medical, Netherlands) Video Microscope System (No.16A00102) with a universal serial bus (USB) 3 port to obtain sidestream dark-field imaging along with the AVA 4.3C software (MicroVision Medical) to evaluate the microcirculation images. We performed the microcirculatory assessment based on the second consensus on the assessment of sublingual microcirculation in critically ill patients compiled by the ESICM task force. We took PPV values and De Backer density of large and small vessels for analysis per the second consensus on the assessment of sublingual microcirculation guidelines [22]. Appropriate training was given to our investigators who performed the microcirculation assessment. The following parameters of sublingual microcirculation were captured: a) number of small vessels sampled; b) the number of all vessels sampled; c) proportion of PPV Small; d) proportion of all PPV; e) density of vessels (De Backer density); f) density of small vessels (De Backer density Small).

Microcirculation assessment is a new tool, and validated threshold values have yet to be established for this investigation. Various studies have reported different cutoffs of these variables between survivors and non-survivors of sepsis and healthy controls. De Backer et al. evaluated 252 patients and found that the mean PPV Small was 71% (range, 65-78) in survivors vs. 50% (range, 40-66) in non-survivors [22]. For this study, we assumed PPV Small cutoff thresholds of 70% and 50%.

Statistical analysis

The study variables were collected in a structured data collection form as continuous or dichotomous variables. The data was entered into a Microsoft Excel spreadsheet (Microsoft Corp., Redmond, WA). We used IBM Corp. Released 2015. IBM SPSS Statistics for Windows, Version 23.0. Armonk, NY: IBM Corp. to analyze the data. We used the chi-square test for dichotomous variables and the student’s t-test for continuous variables. P<0.05 was considered significant.

## Results

Sixty-one cases fulfilled the inclusion criteria and were subsequently screened. Two of them denied consent and were excluded. Among the remaining 59 cases, six patients had to be excluded because of an inability to give adequate fluid resuscitation (30 ml/kg over three hours) as per management guidelines (i.e., heart failure with reduced ejection fraction: two; chronic kidney disease: two; chronic liver disease: one; severe anemia: one). Two participants had to be excluded because of equipment failure. We could finally analyze 51 cases. Eight patients ultimately required inotrope, and six patients died.

The mean age of the patients was 41.0 ± 14.9 years, with a slight female predominance of 60.8% (n=31) with 39.2% males (n=20). All cases were recruited from emergency departments (35.3%) or various medical wards (64.7%). The most common suspected focus of infection was gastrointestinal (33.3%), followed by respiratory infections (25.5%), genitourinary (11.8%), skin and soft tissue infections (3.9%), and meningoencephalitis (2%). Undifferentiated tropical fever accounted for (23.5%) of the cases. The mean duration of illness was 6.6 ± 3.3 days (range, one to 14 days). Twenty-six patients (50.9%) had tachycardia with a>100 beats per minute pulse rate at baseline. Twenty-five patients (49%) had SBP <90 mmHg. The respiratory rate was ≥22 breaths per minute in 94.1% of cases. Only two patients had a GCS score of ≤14. Seven patients (13.7%) had partial pressure of oxygen ≤ 95%. For all cases, the qSOFA score was ≥2. Details of baseline clinical parameters are provided in Table 1.

**Table 1:** Baseline clinical parameter of all participants (n=51) SD: standard deviation, SBP: systolic blood pressure, DBP: diastolic blood pressure, GCS: Glasgow coma scale, TLC: total leucocyte count, TPC: total platelet count, AST: aspartate aminotransferase, ALT: alanine aminotransferase, SpO2: peripheral saturation via pulse oximeter, PPV: proportion of perfused vessels, PPV Small: proportion of perfused vessels small, De Backer Density: De Backer Density of vessels, De Backer Density Small: De Backer Density of vessels small.

Variables	Mean ± SD
Clinical parameters on admission	
Pulse Rate	97.6 ± 18.3 beats per minute
Respiratory Rate	24.3 ± 3.5 breaths per minute
SBP	89.0 ± 7.0 mmHg
DBP	57.7 ± 8.3 mmHg
GCS	14.8 ± 0.8
SpO2	95.8 ± 6.5%
Arterial Blood Gas analysis	
PaO2	96.0 ± 23.2 mm Hg
HCO3	19.9 ± 4.4 mEq/L
Lactate	1.4 ± 0.7 mmol/L
PaCO2	27.9 ± 6.0 mm Hg
P/F Ratio	451.2 ± 121.0
pH	7.4 ± 0.0
Sepsis Scores	
qSOFA	2.0 ± 0.1
SOFA Score	2.4 ± 2.0
Complete Blood count parameters	
TLC	10697.2 ± 9341.4 per mm^3^
TPC	268180.3 ± 200248.5 per mm^3^
Hemoglobin	11.1 ± 2.3 g/dL
Liver Function Test	
ALT	64.2 ± 94.7 U/L
AST	96.4 ± 148.2 U/L
Total Bilirubin	1.4 ± 3.9 mg/dL
Kidney function test	
Serum Creatinine	0.9 ± 0.6 mg/dL
Potassium	3.9 ± 0.7 mEq/L
Sodium	131.1 ± 8.2 mEq/L
Microcirculatory variables	
PPV	79 ± 14%
PPV Small	60 ± 19%
De Backer Density	3.81 ± 1.03 n/mm
De Backer Density Small	1.08 ± 0.84 n/mm

Of our 51 individuals, 10 patients had a lactate value >2 mmol/L (19.6%). Hyponatremia (i.e., sodium value <135 mmol/L) was found in 33 of 51 patients (64.7%). Regarding the SOFA score variables, the partial pressure of oxygen to fraction of inspired oxygen ratio was <300 in six individuals (11.7%), while 15 (29.4%) individuals had a total platelet count of <150 x103/µL. Nine individuals (17.6%) had a total bilirubin of >1.2 mg/dL while 10 individuals (19.6%) had elevated creatinine values (>1.2 mg/dL). The SOFA score was ≥3 in 22 individuals (43.1%).

Thirty of 51 cases (58.8%) had a PPV Small value of ≤70% at baseline. A PPV Small value of ≤50% was found in 12 of 51 cases (23.5%). Eight patients required inotropes at the end of the study period (24 hours).

The individuals who required inotrope were younger (36.1 ± 13.5 years) than those who did not require inotrope (41.9 ± 15.2 years), but this was not statistically significant. Eighty percent of men and 87.1% of women did not require inotrope, and there was no statistically significant difference in inotrope need according to sex. The mean duration of illness at presentation to the hospital was slightly longer in the inotrope-requiring group than in those who did not require inotrope (6.1 ± 2.9 days vs. 6.7 ± 3.4 days) but without any statistical significance. Lactate levels were significantly higher in the inotrope-requiring group (2.1 ± 1.0 mmol/L) than in the non-inotrope requiring group (1.33 ± 0.6 mmol/L), as noted in Table 2.

**Table 2:** Difference in baseline parameters of inotrope-requiring and non-inotrope-requiring patients SD: standard deviation, SBP: systolic blood pressure, DBP: diastolic blood pressure, GCS: Glasgow coma scale, TLC: total leucocyte count, TPC: total platelet count, AST: aspartate aminotransferase, ALT: alanine aminotransferase, SpO2: peripheral saturation via pulse oximeter, PPV: proportion of perfused vessels, PPV Small: proportion of perfused vessels small, De Backer Density: De Backer Density of vessels, De Backer Density Small: De Backer Density of vessels small.

Variables	Inotrope Requiring group (n=8), mean ± SD	Non-inotrope Requiring group (n=43), mean ± SD	P-value
Clinical parameters on admission			
Pulse rate (beats per minute)	105.0 ± 23.6	96.26 ± 17.1	0.219
Respiratory Rate (breaths per minute)	27.50 ± 5.6	23.81 ± 2.7	0.006
SBP (mmHg)	83.0 ± 7.6	90.24 ± 6.3	0.006
DBP (mmHg)	52.63 ± 6.3	58.74 ± 8.3	0.05
GCS	15.0 ± 0.01	14.81 ± 0.9	0.588
SpO2 (%)	92.0 ± 14.5	96.5 ± 3.3	0.068
Arterial Blood Gas analysis			
PaO2 (mm Hg)	102.65 ± 21.0	94.7 ± 23.6	0.385
HCO3 (mEq/L)	19.92 ± 2.2	20.0 ± 4.7	0.962
Lactate mmol/L	2.14 ± 1.0	1.33 ± 0.6	0.006
PaCO2 (mm Hg)	27.65 ± 4.4	28.05 ± 6.3	0.872
P/F Ratio	484.38 ± 92.3	445.06 ± 125.6	0.404
pH	7.45 ± 0.05	7.44 ± 0.05	0.692
Sepsis Scores			
qSOFA	2.0 ± 0.1	2.02 ± 0.1	0.174
SOFA	2.0 ± 1.6	2.51 ± 2.1	0.525
Complete Blood count parameters			
TLC (per mm^3^)	7472.50 ± 5105.96	11297.20 ± 9858.44	0.292
TPC (per mm^3^)	286625.0 ± 171088.06	264748.84 ± 206838.35	0.780
Hemoglobin (g/dL)	10.93 ± 3.5	11.23	0.752
Liver Function Test			
ALT (U/L)	21.96 ± 19.5	72.46 ± 101.3	0.170
AST (U/L)	37.42 ± 27.1	108 ± 159.41	0.222
Bilirubin (mg/dL)	0.73 ± 0.3	1.63 ± 4.3	0.563
Kidney function test			
Serum Creatinine (mg/dL)	0.86 ± 0.40	1.0 ± 0.68	0.561
Potassium (mEq/L)	4.25 ± 0.9	3.8 ± 0.7	0.174
Sodium (mEq/L)	130.75 ± 4.3	131.27 ± 8.8	0.872
Microcirculatory variables			
PPV (%)	0.76 ± 0.3	0.80 ± 0.1	0.447
PPV Small (%)	0.55 ± 0.1	0.61 ± 0.1	0.459
De Backer Density (n/mm)	3.86 ± 0.8	3.80 ± 1.0	0.888
De Backer Density Small (n/mm)	1.10 ± 0.4	1.08 ± 0.9	0.964

PPV and PPV Small were lower in the inotrope-requiring group than in the non-inotrope-requiring group without any statistical significance. De Backer density and De Backer density Small were higher in the inotrope requiring group than in the non-inotrope requiring group without statistical significance. None of the four microcirculatory variables could predict the requirement of inotrope at the baseline, as noted in Table 3.

**Table 3:** Baseline sublingual microscopy parameters and inotrope requirement CI: confidence interval, PPV: proportion of perfused vessels, PPV Small: proportion of perfused vessels small.

Variables	Unadjusted odd ratio (95% CI)	P-value
PPV	0.037 (0.00-478)	0.49
PPV Small	0.75 (0.002-6.94)	0.92
De Backer Density	1.38 (0.37-5.76	0.65
De Backer Density Small	0.53 (0.04-5.34)	0.51

There was also no significant difference in these microcirculatory parameters among patients who survived compared to those who died.

## Discussion

Most of our participants were recruited from the medical wards (64.7%), and the rest were recruited from emergency departments. Rasmy et al. from Egypt, De Backer et al. from Belgium, Edul et al. from Argentina, and most similar studies were conducted in intensive care unit (ICU) settings [24-26]. Our study more closely resembled the patient population encountered in routine clinical practice.

We observed that 57.6% of patients were female, and the mean age was 41.0 ± 14.9 years. Shapiro et al. and Rasmy et al. had predominantly older male sepsis patients [24,27]. In Shapiro et al.’s study, the patients with sepsis had a mean age of 55, whereas those with septic shock were older, with a mean age of 68 years. They evaluated individuals presenting to the emergency department, and they had more participants and broader inclusion criteria [27]. Rasmy et al.’s study population had a mean age of 50 years, but the mean age was 54 years in the vasopressor requiring group and 45 years in the non-vasopressor requiring group [24]. The female predominance found in our study probably reflects the regional differences in health-seeking behavior.

The most common suspected foci of infection were gastrointestinal (33.3%), followed by respiratory (25.5%), and undifferentiated tropical fever (23.5%). This finding is consistent with other studies on sepsis carried out in other parts of the world [25]. Edul et al.’s study in an Argentinian population found that the most common sources of sepsis were abdominal and respiratory, followed by genitourinary infections [25].

Among the clinical parameters, the mean pulse was on the higher side (mean 97.6 ± 18.3 beats per minute), and the respiratory rate was also on the higher side (24.3 ± 3.5 breaths per minute). Our inclusion criteria can explain the lower SBP and DBP: we recruited patients with SBP <100 mmHg but MAP >55 mmHg. The rise in pulse rate is self-explanatory, being a compensatory mechanism to maintain homeostasis in the event of low blood pressure. The fever may also explain the higher pulse rate in these infectious syndromes. Lara et al. found their mean heart rate was also high (110 ± 21 beats per minute), similar findings to our study [28]. The mean SOFA score in our patients was 2.4 ± 2.0, and the mean qSOFA was 2.0 ± 0.1. Lara et al. reported that their mean SOFA score was four [28]. Most of the earlier studies were conducted on established sepsis groups, with the individual participants having higher baseline SOFA and qSOFA values. However, as we recruited only early sepsis patients, the mean SOFA and qSOFA values were lower, establishing that our patient population was less critical and more closely resembled real-world scenarios rather than only including severe sepsis cases in high-priority areas like the ICU.

In our study, eight cases required inotrope (15.68%) at the end of the observation period (24 hours). As this is a first-of-its-kind study, there were no studies in the literature against which we could compare our results. The individuals who required inotrope were younger (36.1 ± 13.5 years) than the non-inotrope requiring group (41.9 ±15.2 years), with an equal distribution between the sexes and a shorter duration of illness (6.1 ± 2.9 days vs. 6.7 ± 3.4 days). Rasmy et al. found that 21 of 36 patients (58.3%) required vasopressors, and the patients who required vasopressors were older (mean age, 54 years) compared to the non-vasopressor requiring group (mean age, 45 years). Rasmy et al. recruited participants from the ICU, where patients are usually more critical and older. These individuals had more severe diseases than our study participants, explaining the higher percentage of vasopressor use [24].

The initial DBP and SBP were lower in the inotrope requiring group (52.6 ± 6.3 mmHg vs. 58.7 ± 8.3 mmHg and 83.0 ± 7.6 mmHg vs. 90.2 ± 6.3 mmHg, respectively), which is self-explanatory. The mean pulse rate was higher in the inotrope requiring group than in the non-inotrope group (105.0 ± 23.6 beats per minute vs. 96.2 ± 17.1 beats per minute). Lactate levels were higher in the inotrope requiring group (2.1 ± 1.0 mmol/L vs. 1.3 ± 0.6 mmol/L). Rasmy et al. found similar differences in blood pressures, heart rates, and lactate levels between vasopressor requiring vs. non-vasopressor requiring groups [24].

In our study, the mean PPV was 79%, and the mean PPV Small was 60%. De Backer et al. found that the PPV Small was 71% in survivors vs. 50% in non-survivors [26]. We found that 58.8% of our participants had a PPV Small value of ≤70%, while 23.5% had a PPV Small value of ≤50%. The mean De Backer Density in our study was 3.81 ± 1.03, while the De Backer Density Small was 1.08 ± 0.84 (1.10 ± 0.40 in the inotrope requiring group vs. 1.08 ± 0.90 in the non-inotrope requiring group). De Backer et al. found that the density of perfused small vessels was 3.4 n/mm in survivors vs. 2.2 n/mm in non-survivors [26]. We found no other study that demonstrated De Backer Density in a group of patients like ours. Two microcirculatory variables at baseline were lower in the inotrope requiring group. PPV pre-fluids in the inotrope requiring group were 76%, while it was 80% in the non-inotrope requiring group. PPV Small pre-fluids was 55% in the inotrope requiring group and 61% in the non-inotrope requiring group. These findings were expected and aligned with the results from De Backer et al. [26]. De Backer et al. found that PPV Small was significantly reduced in patients with severe sepsis (48%, range, 33%-61%) compared to the control group (90%, range, 89%-92%) [29]. The PPV Small data in our study were consistent with the studies mentioned above. Like us, Massey et al. found no significant difference in De Backer densities between the two groups [30].

Our study was conducted on patients with early sepsis, and the recruitment was done from both high- and low-priority areas. Therefore, the individuals in our study represented the patients we encounter on a day-to-day basis. SOFA, qSOFA score, and lactate levels were available at baseline, making the dataset more robust. However, our study was limited by its low sample size. Therefore, we could not define predictive cutoff values for the microcirculatory variables to predict a poorer prognosis or mortality.

## Conclusions

Sublingual microcirculatory assessment with HVMs is a new and exciting entry in managing patients with sepsis and septic shock. As there are no validated cutoff values of the various microcirculatory parameters in use, it is difficult to differentiate normal and abnormal values. Microcirculatory parameters obtained from sublingual microscopy at initial presentation could not predict the ultimate use of inotrope in patients presenting with early sepsis. There was a significant association of a high respiratory rate and high serum lactate values with a poorer prognosis, but microcirculatory values could not predict a poor outcome, according to our results.
